# Supplementary material for: Mechanism of transcription modulation by the transcription-repair coupling factor
Source: Nucleic Acids Res. 2022 May 31;50(10):5688–712. doi: 10.1093/nar/gkac449 (PMC9177983; doi:10.1093/nar/gkac449)
Supplement: gkac449_Supplemental_File [file gkac449_supplemental_file.pdf]

SUPPLEMENTARY INFORMATION

Mechanism of transcription modulation by the transcription-repair coupling factor

Bishnu P Paudel[1,2], Zhi-Qiang Xu[1,2], Slobodan Jergic[1,2], Aaron J Oakley[1,2], Nischal Sharma[1,2], Simon HJ Brown[1,2,3], James C Bouwer[1,2,3], Peter J Lewis[1,4], Nicholas E Dixon[1,2,3], Antoine M van Oijen[1,2,3] and Harshad Ghodke[1,2]*

[1]Molecular Horizons and School of Chemistry and Molecular Bioscience, University of Wollongong, Wollongong, NSW 2522, Australia

[2]Illawarra Health and Medical Research Institute, Wollongong, NSW 2522, Australia

[3]ARC Industrial Transformation Training Centre for Cryo-electron Microscopy of Membrane Proteins, University of Wollongong, Wollongong, NSW 2522, Australia

[4]School of Environmental and Life Sciences, University of Newcastle, Callaghan, NSW 2308, Australia

* To whom correspondence should be addressed. Tel: +61 2 4239 2371; Email: harshad@uow.edu.au

Present Address: Harshad Ghodke, Molecular Horizons and School of Chemistry and Molecular Bioscience, University of Wollongong, Wollongong NSW 2522, Australia

Supplemental Figure 1

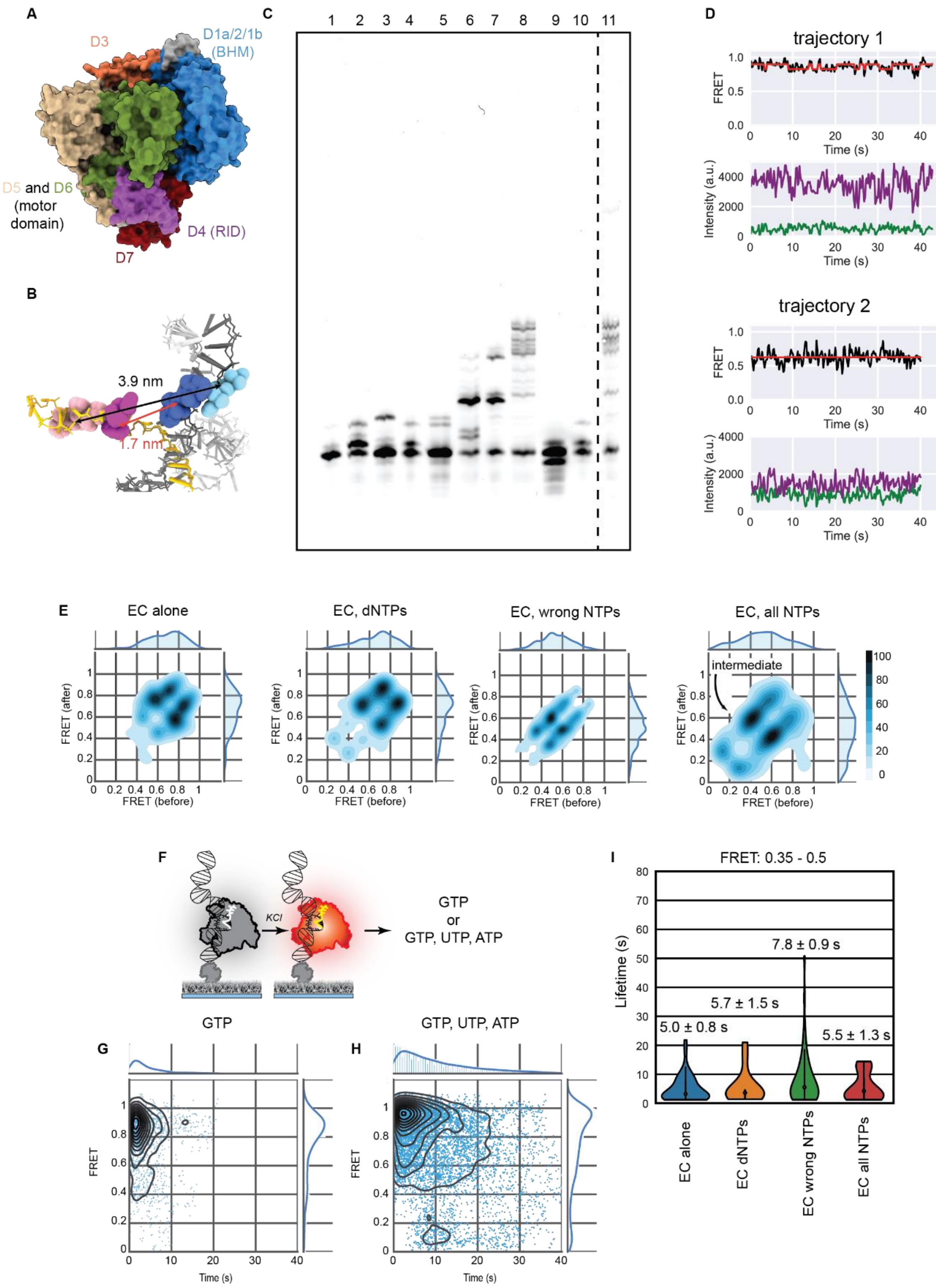

Supplemental Figure S1: Walking the EC with subsets of correct rNTPs. *Related to Figure 1.*

(A) Crystal structure of apo Mfd (PDB 2EYQ) colored by functional modules: UvrB homology module (blue), domain 3 (coral), domain 4 (RNAP interacting domain; purple), motor domains 5 (tan) and 6 (olive), auto-inhibitory domain 7 (dark red).

(B) Schematic of the *E coli* transcription bubble (non-template: light gray, template: dark gray, RNA: gold) with label positions highlighted. Blue spheres represent Cy3 and magenta/pink spheres represent Cy5 inserted in the backbone of the nucleic acids. The distances between the cyanine dyes are indicated for the pre-translocated register (1.7 nm) and post-translocated register (3.9 nm) (see also Supplemental Note 1).

(C) Denaturing gel showing nucleotide incorporation and pyrophosphorolytic activity of the EC. ECs were reconstituted on magnetic Ni-NTA beads (see Methods) and tested for catalytic activity. Lane 1: EC alone or with 100 μM of GTP (Lane 2), UTP (Lane 3), ATP (Lane 4) and CTP (Lane 5), GTP + UTP (Lane 6), GTP + UTP + ATP (Lane 7) and all NTPs (Lane 8). EC incubated with PPi (1 mM) (Lane 9), ATP (1 mM) (Lane 10), and ATP (1 mM) and PPi (1 mM) followed by all NTPs (100 μM each) (Lane 11).

(D) Two example FRET trajectories of the EC alone (as shown in Figure 1B) and corresponding intensity traces for the Cy3 donor signal (green) and Cy5 acceptor (magenta). Trajectories are truncated at the photobleaching step.

(E) Transition density plots for RNAP alone (*n* = 318 molecules), in the presence of dNTPs (*n* = 79 molecules), with non-complementary ('wrong') NTPs (*n* = 179 molecules) and the full set of NTPs (*n* = 81 molecules) respectively.

(F) Schematic of *in situ* assembled FRET-pair labeled ECs incubated with GTP (the next correct NTP) (*n* = 63 molecules) or with GTP, ATP and UTP (*n* = 87 molecules). Temporal heat maps of the reaction for experiments conducted in the presence of GTP (G) or GTP, ATP and UTP (H) are presented here.

(I) Lifetimes of FRET intermediates observed in indicated conditions in the FRET range from 0.35 to 0.5. See Supplemental Note 2 and Supplemental Table 2.

Supplemental Figure 2

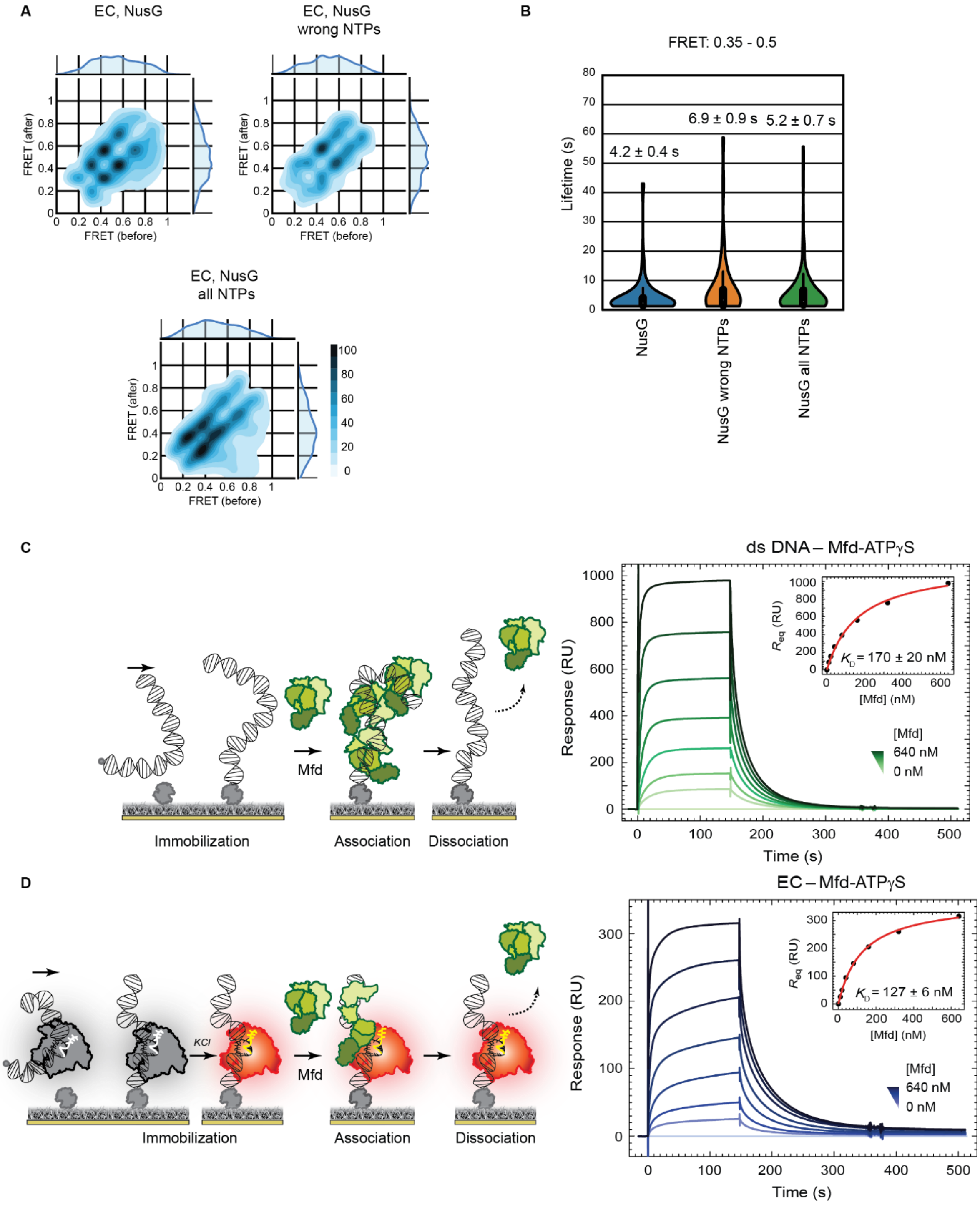

Supplemental Figure S2: Measurement of binding affinity of Mfd for dsDNA and the EC. ***Related to Figures 2 and 3.***

(A)Transition density plots for EC and NusG alone (n = 461 molecules), EC and NusG in the presence of wrong NTPs (n = 231 molecules), and EC and NusG with the full set of NTPs (n = 426 molecules).

(B) Lifetimes of FRET intermediates observed in indicated conditions in the FRET range from 0.35 to 0.5. See Supplemental Note 2 and Supplemental Table 2.

(C, D) Schematic and SPR sensorgrams show association (150 s) and dissociation phases of serially-diluted 10–640 µM Mfd-ATPγS, including a 0 µM control for (C) biotinylated 49-mer dsDNA, and (D) *in vitro* reconstituted EC on biotinylated 49-mer DNA flowed into the SPR chip. Responses at equilibrium averaged over the steady region of sensorgrams (R_{eq}) were fit (insets) using a steady-state affinity (SSA) model (Equation 1, Materials and Methods) to derive values of K_D (as indicated). Errors are S.E. of the fit.

Supplemental Figure 3

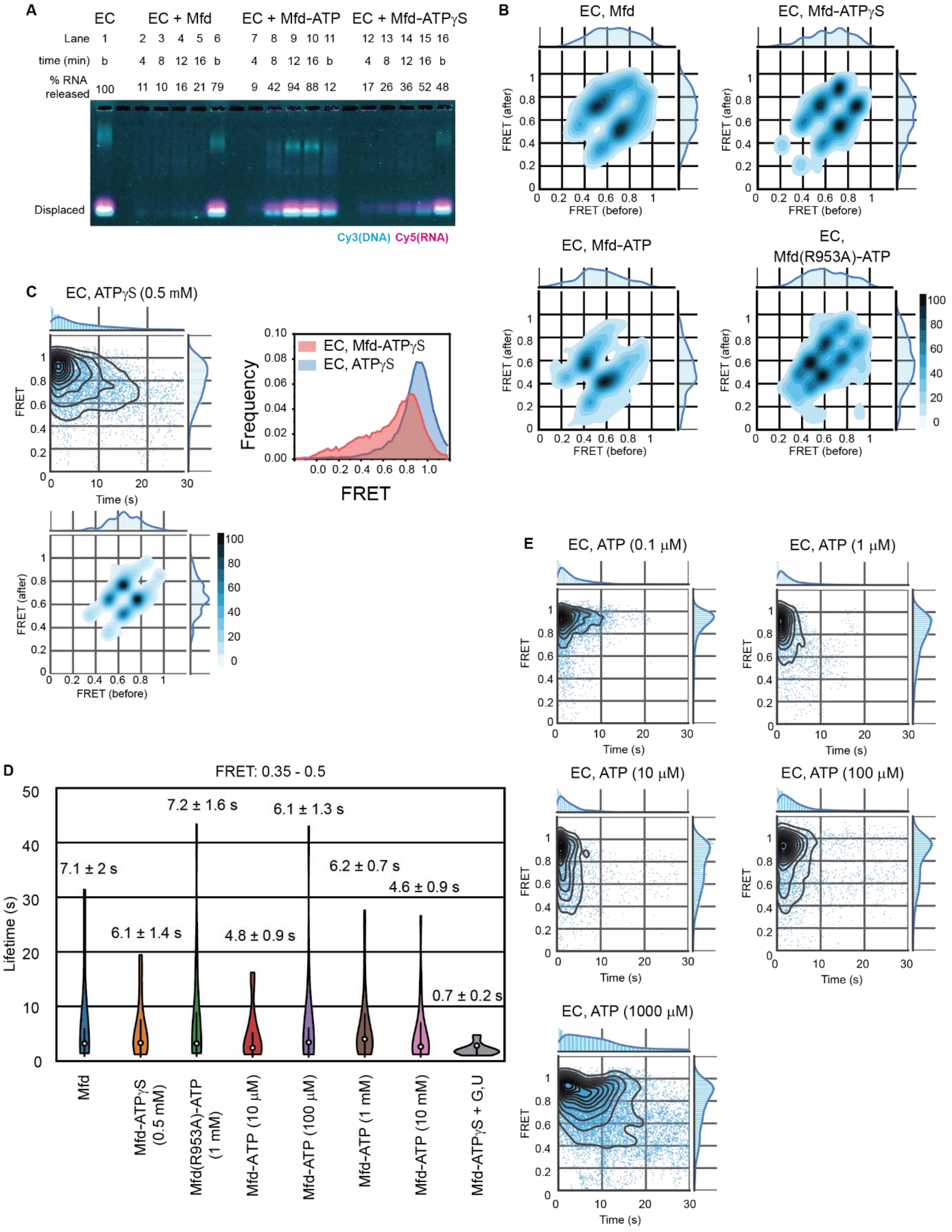

Supplemental Figure S3: Interactions of ATP with the EC alone or in the presence of Mfd. *Related to Figure 3.*

(A) Overlay of agarose gel showing DNA (Cy3, cyan) and RNA (Cy5, magenta) reaction products from time course of Mfd-mediated transcription termination. Reconstituted, unreacted ECs from the bead fraction are shown in lane 1. Supernatant fraction was sampled from tubes containing ECs incubated with Mfd alone (lanes 2, 3, 4, 5) or with Mfd (500 nM) and 0.5 mM ATPɣS (lanes 6, 7, 8, 9) or with Mfd (500 nM) and 1 mM ATP (lanes 10, 11, 12, 13) at 4, 8, 12 and 16 min respectively. Percentage of total RNA displaced in the supernatant or retained on beads is indicated for each lane. Note that the migration of the free RNA is different from that of free template. Mfd efficiently displaces transcript and DNA from the elongation complex in the presence of ATP compared to ATPɣS or in the absence of ATP.

(B) Transition density plots for EC and Mfd alone (*n* = 75 molecules), EC and Mfd in the presence of 1 mM ATPɣS (*n* = 68 molecules), EC and Mfd in the presence of 1mM ATP (*n* = 174 molecules), and EC and Mfd(R953A) in the presence of 1 mM ATP (*n* = 234 molecules).

(C) Temporal heat map (upper panel) and transition density plot (lower panel) of FRET pair labelled RNAP EC in the presence of 0.5 mM ATPɣS alone (*n* = 124 molecules). For convenience, the ensemble FRET distribution of the sample of EC incubated with ATPɣS is directly compared to that incubated with Mfd-ATPɣS (Figure 3C) in a single plot.

(D) Lifetimes of FRET intermediates observed in indicated conditions in the FRET range from 0.35 to 0.5. See Supplemental Note 2 and Table 2.

(E) Temporal heat maps of FRET pair labelled RNAP EC when titrated with ATP in the range of 0.1 µM to 1 mM (*n* = 151, 82, 81, 76, 157 molecules respectively) as indicated.

Supplemental Figure 4

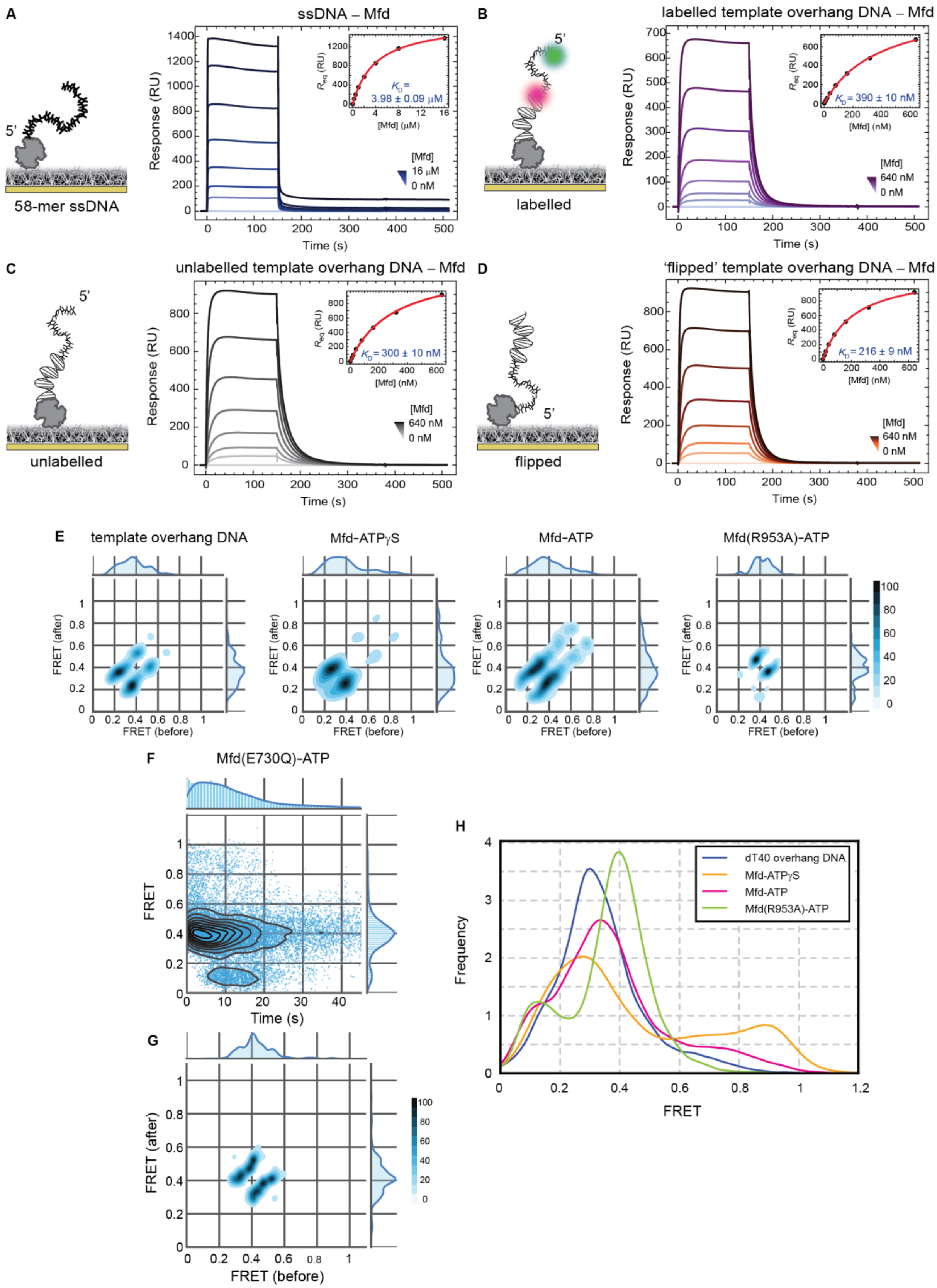

Supplemental Figure S4: Interactions between Mfd and a primed-DNA template. ***Related to Figure 4.***

Schematic and SPR sensorgrams show association (150 s) and dissociation phases of serially-diluted 10–640 µM Mfd-ATPγS, including a 0 µM control for (A) 58-mer ssDNA containing the dT_{40} sequence, (B) FRET pair labeled primed DNA substrate (biotinylated 18-mer dsDNA with dT_{40} overhang), (C) primed DNA substrate (biotinylated 18-mer dsDNA with dT_{40} overhang) and (D) "flipped" primed DNA substrate (18-mer dsDNA with biotinylated dT_{40} overhang). Responses at equilibrium averaged over the steady region of sensorgrams (R_{eq}) were fit (insets) using a steady-state affinity (SSA) model to derive values of K_D (as indicated). Errors are S.E. of the fit.

(E) Transition density plots for FRET-pair labeled primed DNA alone (n = 100 molecules) or bound to Mfd in the presence of ATPγS (n = 94 molecules), or ATP (n = 203 molecules), and Mfd(R953A) in the presence of ATP (n = 105 molecules) are presented here. The DNA substrate is the same as described in Figure 4.

(F, G) Temporal heat map (F) and transition density plot (G) for ATPase mutant Mfd(E730Q) binding to FRET pair labeled primed DNA template used in Figure 4 (n = 103 molecules).

(H) For convenience, kernel density estimations of the ensemble FRET distributions read as projections along the ordinate in the heat-maps presented in Figure 4 are presented to enable direct visual comparisons.

Supplemental Figure 5

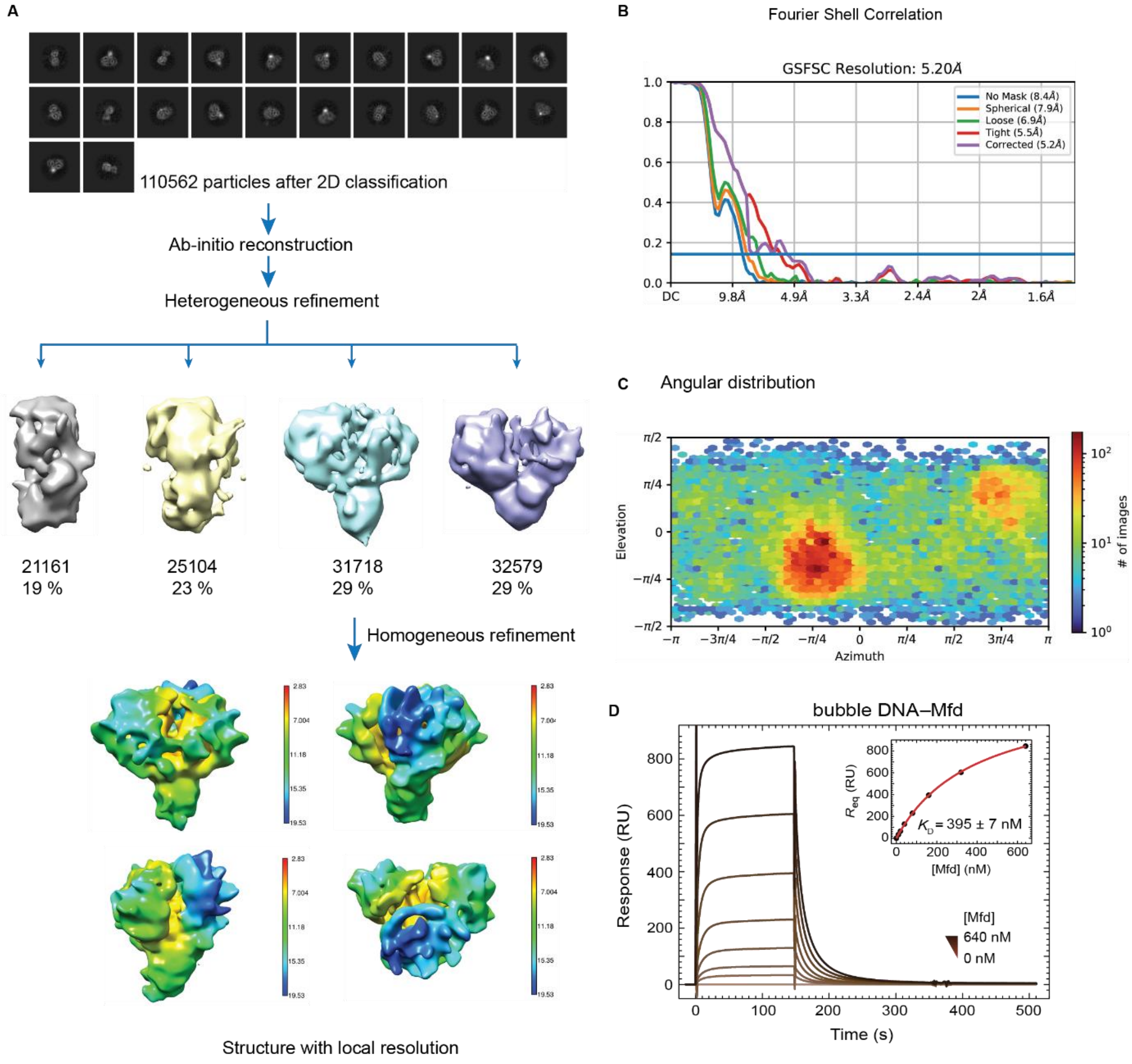

Supplemental Figure S5: EM reconstruction of Mfd. *Related to Figure 5.*

(A) Overview of image processing using CryoSPARC. A set of 110562 particles were selected after 2D classification for *ab initio* reconstruction and heterogeneous refinement leading to four classes of 3D reconstructions. Of these, 31718 particles that yielded the most complete reconstruction were subjected to homogenous refinement to generate the map presented here. The colormap applied to the reconstruction represents local resolution.

(B) Fourier shell correlation curves of the reconstruction reported an apparent resolution of 5.2 Å (gold-standard 0.143 FSC).

(C) Angular distribution of particle projections

(D) Measurement of the dissociation constant of Mfd for a 49-mer dsDNA template containing a 9 nucleotide non-complementary bubble where the complementary residues were replaced by dT_9. The errors reflect S.E. of the fit.

Supplemental Note 1

To assess whether the calculated distances are consistent with the FRET state assignments for the pre- *vs*. post- translocated states, we built an *in silico* model (Supplemental Figure 1B) based on available structures of the *E. coli* elongation complex (1) and structures of the Cy3 and Cy5 dyes (2). The distances between the Cy3 and Cy5 dyes in the pre- and post-translocated states were then estimated to be 1.7 and 3.9 nm, respectively. Using these distances, and available estimates for R_0 for Cy3–Cy5 dye pairs introduced into protein-DNA complexes in the range of 4.7–6.5 nm (3), we estimate FRET efficiencies for the pre- and post-translocated states to be: 0.99 and 0.75 (for R_0 = 4.7 nm) and 0.99 and 0.95 (for R_0 = 6.5 nm). The measured FRET efficiencies are ~0.9 and ~0.6 (Figure 1B–E), which agree well with the lower estimates for R_0.

Supplemental Note 2

To measure the lifetime of the intermediate state we first chose to analyze trajectories with intermediates in the mid-FRET regime. However, examination of individual trajectories revealed that this mid-FRET regime is populated by molecules exhibiting high-mid FRET state dynamics as well as mid-low FRET dynamics. We therefore selected molecules in the 0.35–0.5 FRET range which mostly accommodated molecules exhibiting mid-low FRET dynamics. The mean lifetimes (and standard error) of the intermediates states were best estimated by fitting the distributions to single-exponential fits. In the case of one notable condition – Mfd in the presence of ATPɣS followed by incubation with correct nucleotides – the lifetime distributions was found to be better fit by a gamma distribution. In this case, the scale parameter was used to report the lifetime (Supplemental Table 2).

Supplemental Note 3

The nucleotide-starved EC elongates the transcript by misincorporation of ATP and ATPɣS at millimolar concentrations of these nucleotides within 10 minutes (Supplemental Figure 1C). We confirmed that transcript elongation through misincorporation of AMP occurs in the presence of Mfd under the conditions used for probing the binding and catalytic activities of Mfd in bulk assays (data not shown). Unlike the bulk Mfd assays, observation of Mfd activity in smFRET investigations in the presence of ATP and ATPɣS was limited to the first two movies (a total of four minutes) to capture events that occur rapidly upon introduction of Mfd-ATP/ATPɣS into the flow chamber. Since incorporation of cognate NTPs leads to a drop in FRET in this assay, we wondered whether misincorporation of AMP at mM

concentrations of ATP or ATPɣS explains the drop in FRET signal observed in the presence of these nucleotide cofactors. Therefore, we compared ensemble FRET histograms of the EC incubated with ATPɣS (0.5 mM) alone or in the presence of Mfd and ATPɣS (Supplemental Figure 3C; right panel). Within the relevant observation window of four minutes the FRET efficiency distribution of the EC-ATPɣS sample exhibited a major peak at 0.92, distinct from the EC-Mfd-ATPɣS sample which showed a clear shift in the major peak to lower FRET efficiency of 0.8 and the significant presence of molecules exhibiting additional lower FRET efficiency states. Thus, misincorporation of AMP into the transcript in the presence of ATPɣS does not explain the lower-FRET populations observed in the presence of Mfd-ATPɣS (or ATP). A unique signature for the misincorporated EC-AMP complex from the EC complex alone was not detected on this timescale suggesting either inefficient incorporation or (likely) a reorganization of the complex resulting in the same FRET efficiency.

Supplemental Note 4

To compare the efficiency of transcription by the elongation complex following incubation with Mfd-ATPɣS upon addition of 100 µM each of GTP and UTP to that in the presence of GTP, UTP and ATP, we plotted histograms of the FRET states observed in the system (gray bars in Figure 3L). The histogram for each condition was best fit with a sum of three Gaussian terms of the form:

$$f(x) = ae^{-\frac{(x-\mu)^2}{2\sigma^2}}$$

Here a_i represents the amplitude, μ_i represents the mean of the distribution and σ_i the standard deviation of the ith distribution. In the absence of Mfd-ATPɣS, the three Gaussians (a_i, μ_i, σ_i) were: (25.36, 0.91, 0.11); (16.5, 0.63, 0.33) and (15.14, 0.09, 0.10) with an R-square of 0.88. In the presence of Mfd-ATPɣS and rGTP, rUTP, the three Gaussians were: (17.15, 0.93, 0.19), (10.94, 0.70, 0.27) and (9.82, 0.18, 0.10) with an R-square of 0.85. For each Gaussian the area was calculated as:

$$A_i = \sqrt{2\pi} a_i \sigma_i$$

Next, each area was normalized by the sum of the areas of the three Gaussians to obtain the fractional area occupied by each Gaussian. Efficiencies were calculated by comparing the fractions of the populations corresponding to each of the Gaussians across the two conditions. Since transcript elongation is reflected in the low-FRET states, we compared the fraction of the population in the Gaussian with the smallest mean FRET.

Supplemental Note 5

SPR measurements of the strength of interaction between Mfd and this DNA substrate in the presence of ATPɣS revealed a dissociation constant that is somewhat higher (K_D = 300 ± 10 nM) than that of dsDNA (170 ± 20 nM; Supplemental Figure 4B–D compared to Supplemental Figure 2C). To monitor changes in the distance between the junction and the 5’ end, we introduced a FRET pair on either end of the ssDNA overhang (Figure 4D). SPR measurements revealed an affinity of 390 ± 10 nM for the binding of Mfd to the FRET-pair labelled substrate (Supplemental Figure 4B).

Supplemental Table 1: Nucleic Acid substrates used in this study

Experiment	DNA	Sequence
pETMCSII_Mfd_F	Cloning primer	GTT TAA TCG GAT CCT AAG GAG GTT AAT TCC CGC TAT GCC TGA ACA ATA TCG TTA TAC G
pETMCSII_Mfd_R	Cloning primer	GGGAGCTCGAATTCTTAAGCGATCGCGTTCTCT
smFRET (Figures 1–3)	Non-template (NT70_bio)	ATC GAG CAA CTA CTC AGA CAG CAC TAC TGC GAC TTA CAG ACA TCG AGA GGG TAA TGG CGA ATA GCA CTG A /3 bioTEG/
smFRET (Figures 1–3)	Template (T70_31Cy3)	TCA GTG CTA TTC GCC ATT ACC CTC TCG ATG T/iCy3/CT GTA AGT CGC AGT AGT GCT GTC TGA GTA GTT GCT CGAT
smFRET (Figures 1–3)	RNA(R15_4 Cy5)	rArUrArU/iCy5/rArU rArUrC rGrArG rArGrG
smFRET (Figure 4)	bio_AS18_Cy5	/5Biosg/ TGG CGA CGG CAG CGA GGC/3Cy5Sp/
smFRET (Figure 4)	Cy3_dT40_S18	/5Cy3/TT TTT TTT TTT TTT TTT TTT TTT TTT TTT TTT TTT TTT TTG CCT CGC TGC CGT CGC CA
SPR (Supplemental Figure 4)	bio_AS18	/5Biosg/ TGG CGA CGG CAG CGA GGC
SPR (Supplemental Figure 4)	dT40_S18	TT TTT TTT TTT TTT TTT TTT TTT TTT TTT TTT TTT TTT TTG CCT CGC TGC CGT CGC CA
SPR (Supplemental Figure 4)	bio_dT40_S18	/Biosg/TT TTT TTT TTT TTT TTT TTT TTT TTT TTT TTT TTT TTT TTG CCT CGC TGC CGT CGC CA
SPR	AS18	TGG CGA CGG CAG CGA GGC

(Supplemental Figure 4)		
SPR (Supplemental Figure 2)	TS_49	TCA GTG CTA TTC GCC ATT ACC CTC TCG ATG T/iCy3/CT GTA AGT CGC AGT AGT G
SPR (Supplemental Figure 2)	NT_49_bio	CAC TAC TGC GAC TTA CAG ACA TCG AGA GGG TAA TGG CGA ATA GCA CTG A /3 bioTEG/
SPR (Supplemental Figure 5)	NT_49_bio_ bubble	CAC TAC TGC GAC TTA CAG ACT TTT TTT TTG TAA TGG CGA ATA GCA CTG A /3BioTEG/
SPR (Supplemental Figure 2)	RNA_15	rArUrAr UrArU rArUrC rGrArG rArGrG
SPR (Figure 4)	S18	GCC TCG CTG CCG TCG CCA
SPR (Figure 4)	dT3_S18	TTT GCC TCG CTG CCG TCG CCA
SPR (Figure 4)	dT6_S18	TTT TTT GCC TCG CTG CCG TCG CCA
SPR (Figure 4)	dT9_S18	TTT TTT TTT GCC TCG CTG CCG TCG CCA
SPR (Figure 4)	dT12_S18	TTT TTT TTT TTT GCC TCG CTG CCG TCG CCA
SPR (Figure 4)	dT15_S18	TTT TTT TTT TTT TTT GCC TCG CTG CCG TCG CCA
SPR (Figure 4)	S18_bio	GCC TCG CTG CCG TCG CCA /3Bio/
SPR (Figure 4)	AS18_dT3	TGG CGA CGG CAG CGA GGC TTT

SPR (Figure 4)	AS18_dT6	TGG CGA CGG CAG CGA GGC TTT TTT
SPR (Figure 4)	AS18_dT9	TGG CGA CGG CAG CGA GGC TTT TTT TTT
SPR (Figure 4)	AS18_dT12	TGG CGA CGG CAG CGA GGC TTT TTT TTT TTT
SPR (Figure 4)	AS18_dT15	TGG CGA CGG CAG CGA GGC TTT TTT TTT TTT TTT
2AP Bulk fluorescence (Figure 5)	dT6_S18_v2_11_2AP	TTT TTT GAC T/i2AmPr/A CAG CCG ACG CGT
2AP Bulk fluorescence (Figure 5)	dT6_S18_v2_12_2AP	TTT TTT GAC TA/i2AmPr/ CAG CCG ACG CGT
2AP Bulk fluorescence (Figure 5)	AS18_v2_2AP	ACG CGT CGG CTG TTA GTC
2AP Bulk fluorescence (Figure 5)	AS18_v2_2AP_bubble	ACG CGT CGG CTG AAA GTC

Supplemental Table 2: Lifetimes of mid-FRET states

	Condition	Distribution	Fit Parameter				
			Mean (τ) [Exp]/ scale parameter (Gamma) (s)	S.E.	Shape	S.E.	log likelihood
1	EC (n_states = 42)	exp	5.0	0.8			–109.9
2	EC dNTPs (n_states = 14)	exp	5.7	1.5			–38.4
3	EC wrong (n_states = 70	exp	7.8	0.9			–213.7
4	EC right (n_states = 19)	exp	5.5	1.3			–51.6
5	NusG (n_states = 99)	exp	4.2	0.4			–242.1
6a	NusG wrong (n_states = 57)	exp	6.9	0.9			–166.9
7a	NusG correct (n_states = 129)	exp	5.9	0.5			–357.0
8	Mfd (n_states = 13)	exp	7.1	2.0			–38.5
9	Mfd-ATPɣS (n_states = 20)	exp	6.1	1.4			–56.1
10	Mfd(R953A) (n_states = 21)	exp	7.2	1.6			–62.3
11	Mfd-ATP [10µM] (n_states = 29)	exp	4.8	0.9			–74.9
12	Mfd-ATP [100µM] (n_states = 23)	exp	6.1	1.3			–64.5
13	Mfd-ATP [1 mM] (n_states = 77)	exp	6.2	0.7			–216.9
14	Mfd-ATP [10 mM] (n_states = 27)	exp	4.6	0.9			–68.1
15	ATPɣS_rG_rU (n_states = 17)	gamma	0.7	0.2	5.20	1.73	–29.6

References

1. Abdelkareem, M., Saint-Andre, C., Takacs, M., Papai, G., Crucifix, C., Guo, X., Ortiz, J. and Weixlbaumer, A. (2019) Structural Basis of Transcription: RNA Polymerase Backtracking and Its Reactivation. *Mol Cell*, **75**, 298-309 e294.
2. Liu, Y. and Lilley, D.M.J. (2017) Crystal Structures of Cyanine Fluorophores Stacked onto the End of Double-Stranded RNA. *Biophys J*, **113**, 2336-2343.
3. Levitus, M. and Ranjit, S. (2011) Cyanine dyes in biophysical research: the photophysics of polymethine fluorescent dyes in biomolecular environments. *Q Rev Biophys*, **44**, 123-151.
